# Long Non-Coding RNA Expression Profiling of Mouse Testis during Postnatal Development

**DOI:** 10.1371/journal.pone.0075750

**Published:** 2013-10-10

**Authors:** Jin Sun, Yi Lin, Ji Wu

**Affiliations:** 1 Key Laboratory for the Genetics of Developmental and Neuropsychiatric Disorders (Ministry of Education), Bio-X Institutes, Shanghai Jiao Tong University, Shanghai, China; 2 Key Laboratory of Fertility Preservation and Maintenance of Ministry of Education, Ningxia Medical University, Yinchuan, Ningxia, China; 3 The International Peace Maternity and Child Health Hospital, School of Medicine, Shanghai Jiao Tong University, Shanghai, China; University of Nevada School of Medicine, United States of America

## Abstract

Mammalian testis development and spermatogenesis play critical roles in male fertility and continuation of a species. Previous research into the molecular mechanisms of testis development and spermatogenesis has largely focused on the role of protein-coding genes and small non-coding RNAs, such as microRNAs and piRNAs. Recently, it has become apparent that large numbers of long (>200 nt) non-coding RNAs (lncRNAs) are transcribed from mammalian genomes and that lncRNAs perform important regulatory functions in various developmental processes. However, the expression of lncRNAs and their biological functions in post-natal testis development remain unknown. In this study, we employed microarray technology to examine lncRNA expression profiles of neonatal (6-day-old) and adult (8-week-old) mouse testes. We found that 8,265 lncRNAs were expressed above background levels during post-natal testis development, of which 3,025 were differentially expressed. Candidate lncRNAs were identified for further characterization by an integrated examination of genomic context, gene ontology (GO) enrichment of their associated protein-coding genes, promoter analysis for epigenetic modification, and evolutionary conservation of elements. Many lncRNAs overlapped or were adjacent to key transcription factors and other genes involved in spermatogenesis, such as *Ovol1*, *Ovol2*, *Lhx1*, *Sox3*, *Sox9*, *Plzf*, *c-Kit*, *Wt1*, *Sycp2*, *Prm1* and *Prm2*. Most differentially expressed lncRNAs exhibited epigenetic modification marks similar to protein-coding genes and tend to be expressed in a tissue-specific manner. In addition, the majority of differentially expressed lncRNAs harbored evolutionary conserved elements. Taken together, our findings represent the first systematic investigation of lncRNA expression in the mammalian testis and provide a solid foundation for further research into the molecular mechanisms of lncRNAs function in mammalian testis development and spermatogenesis.

## Introduction

The mammalian testis is the site of spermatogenesis and testosterone production, so it plays a central role in the male reproductive system. Spermatogenesis is the primary biological process in the testis and produces mature haploid spermatozoa from diploid spermatogonia. This developmental process is complicated, and involves a series of cellular differentiation and cell biological events, including spermatogonial proliferation, meiosis of spermatocytes and morphological changes of round spermatids [1], [2]. Elucidation of the molecular mechanisms underlying spermatogenesis is important for our understanding of the genetic regulation of normal male germ cell development. Importantly, this understanding can also direct strategies for clinical diagnosis and therapy of male infertility. Therefore, investigations into the molecular mechanisms of testis development and spermatogenesis are prominent in the field of reproductive biology. To date, these investigations have largely focused on the role of protein-coding genes and small non-coding RNAs, including microRNAs (miRNAs) and piwi-interacting RNAs (piRNAs). The unprecedented advances in high-throughput technologies, such as microarray screening and transcriptome sequencing, have delivered significant advances in the exploration of testis development and spermatogenesis. So far, gene expression profiling, proteome profiling, miRNA profiling, piRNA profiling during testis development or spermatogenesis in mouse have been investigated [3]–[7].

Long non-coding RNAs (lncRNAs) represent a novel class of regulatory molecule, which are arbitrarily defined as transcripts of more than 200 nucleotides (nt) in length that lack significant open reading frames [8]. Advances in genome-wide analyses of the mammalian transcriptome have revealed lncRNAs as a major class of transcript, that is pervasively transcribed [9]. Most lncRNAs are transcribed by RNA polymerase II and possess a 5’ methyl cap as well as a polyadenylated tail, similar to protein-coding mRNAs [10]. Numerous lncRNAs are expressed in a tissue/cell type-specific pattern or in a developmental stage-specific manner [11]–[13]. However, they exhibit very low sequence conservation compared to protein coding genes [14], prompting some to argue that lncRNAs may simply be “transcriptional noise” [15]. Nevertheless, accumulating evidence indicates that lncRNAs are not the "dark matter" of the genome, but that they play significant regulatory roles in various biological processes, including X-inactivation, genomic imprinting, cell differentiation, cell apoptosis, stem cell pluripotency, brain development, retina development, nuclear trafficking, heat shock response, and genome rearrangement [16], [17]. In addition, lncRNAs are also associated with a variety of diseases, such as cancer, neurological disorders, heart disease, and autoimmune disorders [18]. Surprisingly, the mechanisms of action of lncRNAs are so diverse that they can modulate gene expression at multiple levels (e.g. transcriptional, post-transcriptional or epigenetic level). For example, lncRNAs can directly interact with a promoter or exon of a target gene, can regulate alternative splicing of pre-mRNA, and the expression of microRNAs and translation. LncRNAs can also modulate protein activity through binding to a target protein and can generate functional small RNAs as precursors. In addition, lncRNAs can mediate epigenetic changes by recruiting chromatin remodeling complexes to specific genomic loci, such as polycomb repressive complex 1 (PRC1), polycomb repressive complex 2 (PRC2), G9a complex, LSD1/CoREST/REST complex, TLS/CBP/p300 complex, and MLL/WDR5 complex [19], [20].

To date, very few studies on the roles of lncRNAs in mammalian testis development and spermatogenesis have been reported. Lee et al. identified a total of 50, 35 and 24 potential lncRNAs from type A spermatogonia, pachytene spermatocytes, and round spermatids, respectively, through searching SAGE data. Candidates were BLAST searched, mapped and RNA secondary structures were compared against various ncRNA databases. They found that levels of some lncRNAs decreased significantly following induction of differentiation by retinoic acid and the levels of several lncRNAs were decreased by more than a 1000-fold [21]. A recent study revealed that an X-linked gene, *Tsx*, located within the X-inactivation center, is actually a new member of the lncRNA family and is abundantly expressed in meiotic germ cells. *Tsx* mutant male mice have smaller testes resulting from pachytene-specific apoptosis, indicating that Tsx performs general functions in male germ cell development [22]. Another recent study found that a lncRNA, *meiotic recombination hot spot locus* (*mrhl*), negatively regulates Wnt signaling in mouse spermatogonial cells through its protein partner Ddx5/p68 [23].

However, our knowledge of the overall expression status of lncRNAs during post-natal development of the mammalian testis is still very limited. To understand roles of lncRNAs in mammalian testis development and spermatogenesis, we first utilized high throughput microarray screening to investigate lncRNA expression profiles of neonatal (6-day-old) and adult (8-week-old) mouse testes. By comparing the lncRNA expression profiles from two developmental stages, we identified differentially expressed lncRNAs. We further examined their genomic context, the gene ontology (GO) enrichment of their associated protein-coding genes, the epigenetic state of their promoter regions and the presence of evolutionary conserved elements. Our data indicate that lncRNAs are likely to play an important role in testis development and spermatogenesis and provide an important foundation for future research in this field.

## Results

### Overview of lncRNA and mRNA profiles in neonatal and adult mouse testes

**Figure 1:**
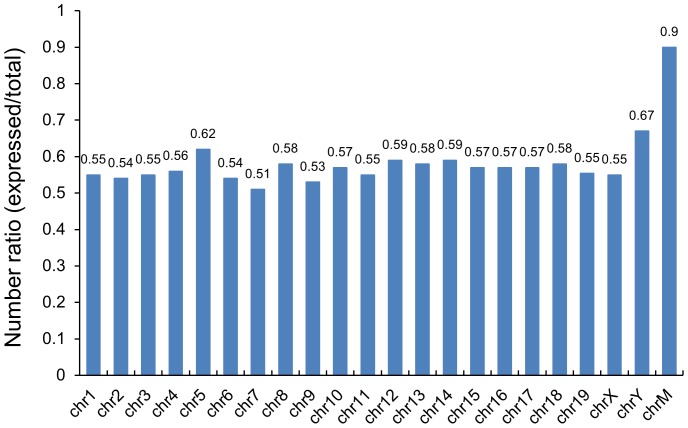
Relative chromosomal distribution of expressed lncRNAs. Chromosomes are on the X axis, and the distribution ratio is on the Y axis. Vertical bands show the ratio (expressed probes/total probes) of expressed lncRNAs derived from each chromosome. “chrM” represents mitochondrial genome.

To examine the lncRNA expression profiles of the mouse testis during post-natal development, we interrogated a commercial mouse lncRNA microarray (stringent version, Arraystar) with RNA isolated from neonatal (6-day-old, N) and adult (8-week-old, A) mouse testes. This microarray contains 14,724 lncRNA probes collected from RefSeq_NR, UCSC_knowngenes, NRED, Fantom 3.0, and 22,635 mRNA probes. The lncRNA probes involve all 20 pairs of chromosome and mitochondrial genome. The overview of lncRNA expression profiles is summarized in Table 1. We found that 56% of lncRNAs on the microarray (8,265 out of 14,724) exhibited expression above background (Table S1), and that 37% of these (3,025 out of 8,265) were significantly differentially expressed (absolute fold-change ≥5; P value ≤0.05) between neonatal and adult mouse testes (Table S2). By contrast, 82% of protein-coding mRNA transcripts on the microarray (18563 out of 22635) were expressed above background (Table S3), and 32% of these (5,964 out of 18,563) were significantly differentially expressed (Table S4). Similar to previous observations [24], [25], our data revealed that a smaller percentage of lncRNAs was detected above background compared to protein-coding genes. This result indicated that lncRNAs exhibited a greater temporal and spatial specificity than protein-coding genes, consistent with previous reports [13], [26]. In addition, statistical analysis showed that lncRNAs expressed above background in mouse testis were widely scattered on all chromosomes (Figure 1, Table S5) and that the ratio (expressed probes/total probes) of lncRNAs expressed from each chromosome was very similar, except for the mitochondrial genome. We inferred that the high relative number of lncRNAs derived from the mitochondrial genome probably relates to the high abundance of mitochondrial lncRNAs in reproductive tissues, such as ovary and testis [27].

**Table 1:** Summary of microarray analysis results.

Probe class	Total	Expressed above background	Differentially expressed*
LncRNAs	14724	8265 (56%)	3025 (37%)
mRNAs	22635	18563 (82%)	5964 (32%)
Combined	37359	26828 (72%)	8989 (34%)

* Significant differential expression was defined as probes with P≤0.05 and absolute fold-change≥5.

### Thousands of lncRNAs are differentially expressed in neonatal and adult mouse testes

**Figure 2:**
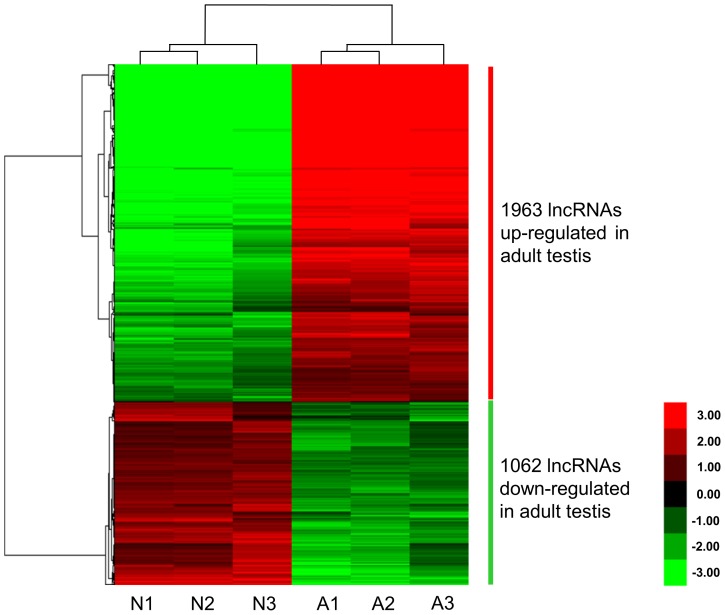
Hierarchical clustering of lncRNAs differentially expressed in neonatal and adult mouse testis. A hierarchical clustered heat map showing the log2 transformed expression values for differentially expressed lncRNAs (absolute fold-change≥5; P≤0.05) between neonatal (N) and adult (A) mouse testis. The intensity of the color scheme is calibrated to the log2 expression values such that red refers to higher transcript abundance and blue refers to lower transcript abundance. The bar code on the right represents the color scale of the log 2 values. Each column represents the data from one of three biological replicates of each sample.

According to the lncRNA expression profiles, 3,025 lncRNAs were differentially expressed (absolute fold-change ≥5; P value ≤0.05) between neonatal (N) and adult (A) mouse testes (Table S2). When we evaluated the expression levels of lncRNAs in paired samples (adult to neonatal ratio, A/N) by log fold-change, 1,062 lncRNAs were found to be significantly down-regulated and 1,963 lncRNAs were significantly up-regulated in the adult testis (Figure 2). The number of up-regulated lncRNAs was almost twice that of down-regulated lncRNAs. In contrast, with regard to the differentially expressed mRNAs (absolute fold-change ≥5), down-regulated mRNAs (3,736 out of 5,964) were more common than up-regulated mRNAs (2,228 out of 5964) (Table S4, Figure S1).

In line with our expectations, some known haploid male germ cell-specific lncRNAs were up-regulated in adult testis, such as *Aldoart2*, *Speer5-ps1* and *Speer9-ps1* [28], [29] (Table 2), and some well-known imprinted lncRNAs were down-regulated, including *H19*, *Meg3*, *Airn* (Table 3). Previous studies have shown that generating the methylation imprint on *H19*, a well-known paternally imprinted gene, is a continuous process during spermatogenesis. *H19* methylation begins between E15.5 and E18.5, but only becomes fully methylated post-natally, by the pachytene spermatocyte stage [30], [31]. It is noteworthy that our data show *H19* to be the most significantly down-regulated lncRNA in the adult testis and this result reaffirms *H19* imprinting in the testis as an incremental process.

**Table 2:** Known haploid male germ cell-specific lncRNAs found in this study.

SeqID	GeneSymbol	FCAbsolute	p-value	Regulation	Literature
NR_003959	Aldoart2	3123.38	2.24E-04	up	Vemuganti et al. 2007
NR_027506	Speer5-ps1	330.46	0.001	up	Spiess et al. 2003
NR_001583	Speer9-ps1	109.92	1.10E-04	up	Spiess et al. 2003

**Table 3:** Known imprinted long noncoding genes found in this study.

SeqID	Gene Symbol	FCAbsolute	p-value	Regulation	Imprinting Type
NR_001592	H19	365.50	0.003	down	paternally imprinting
uc007par	Meg3/Gtl2	39.46	0.008	down	paternally imprinting
NR_002853	Airn	37.44	0.007	down	paternally expression
uc007paq	Meg3/Gtl2	10.14	0.018	down	paternally imprinting
uc007pav	Meg3/Gtl2	9.12	7.17E-04	down	paternally imprinting
uc007pat	Meg3/Gtl2	8.47	0.029	down	paternally imprinting

In addition, we found that only the number of lncRNAs transcribed from the X chromosome is much larger in the down-regulated group than the up-regulated group. In contrast, the number of lncRNAs from all other chromosomes is larger in up-regulated group rather than down-regulated group (Figure S2). And predominant expression of X chromosomal lncRNAs early in spermatogenesis is positively correlated with the greater expression in spermatogonia of protein-encoding genes from the X chromosome[32].

### qRT- PCR results are concordant with microarray data

**Figure 3:**
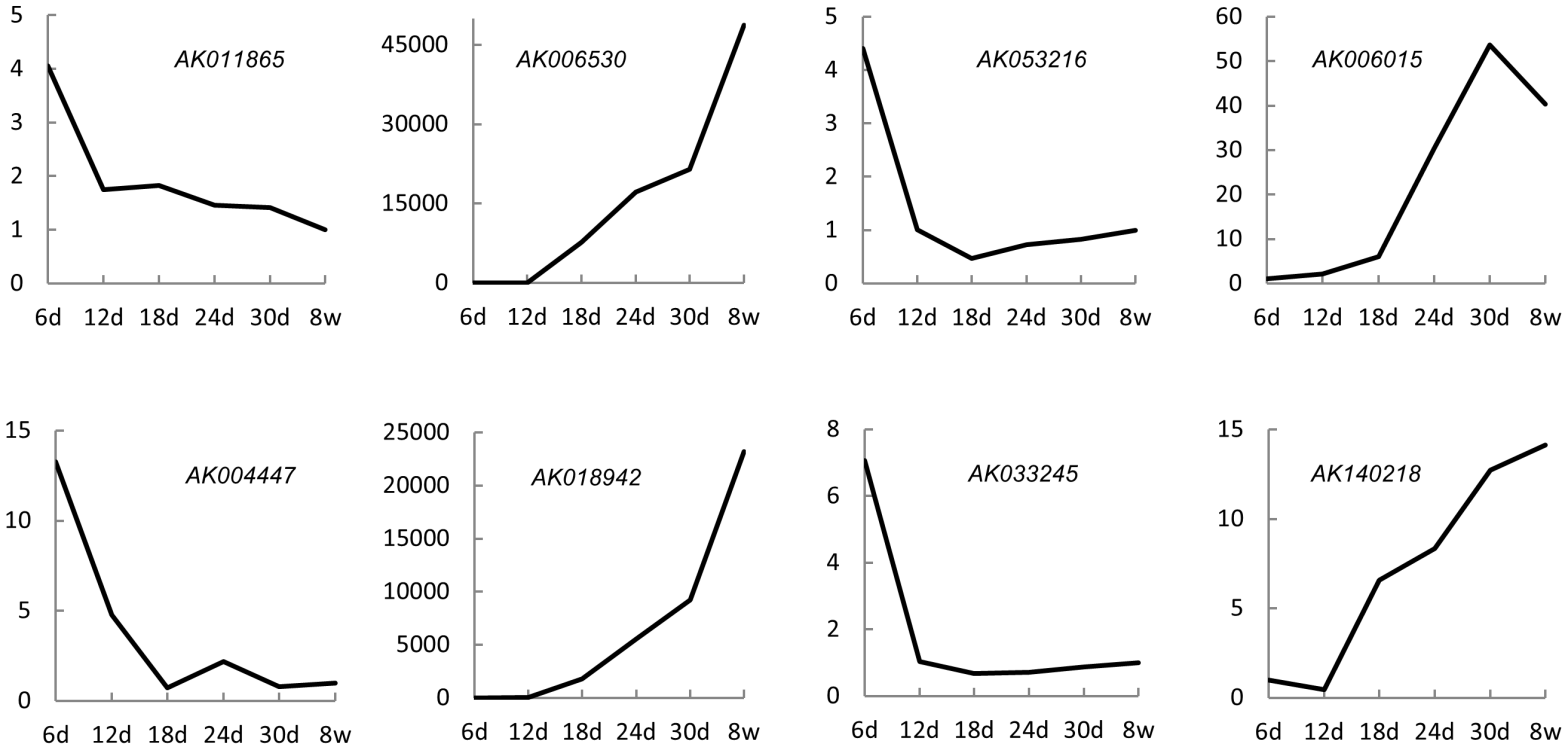
Expression patterns of randomly selected diferentially expressed lncRNAs on different time points of postnatal testis development. The vertical axis indicates relative expression levels of each lncRNA. The relative expression levels were assessed by Q-PCR and were normalized with GAPDH gene. Each result was the average of three independent biological replicates. The horizontal axis indicates the six time points of postnatal testis development from 6 days (d) to 8 weeks (w) after birth.

To validate the microarray data, we investigated the expression patterns of eight randomly selected lncRNAs which identified to be differentially expressed on six time points of postnatal testis development involving 6-day-old, 12-day-old, 18-day-old, 24-day-old, 30-day-old and 8-week-old using quantitative real-time PCR (qRT-PCR). The results clearly showed that expression patterns of these eight selected lncRNAs were concordant with microarray data (Figure 3). Additionally, We found there was a high correlation (Spearman coefficient rho = 0.952, p<0.01, n = 8) between the microarray data and the qRT-PCR data (Table 4). These results demonstrated that the microarray results were reliable.

**Table 4:** Validation of microarray results by qRT-PCR.

	Microarray		qRT-PCR	
LncRNA ID	Fold change a	P value b	Fold change a	P value b
AK011865	–14.700	0.004	–4.056	0.023
AK006530	3718.218	5.11E-04	48767.542	0.017
AK053216	–9.943	0.008	–4.408	0.024
AK018942	14097.462	2.86E-04	23206.382	0.076
AK006015	45.452	2.10E-04	40.391	0.034
AK004447	–40.531	0.002	–13.269	0.028
AK033245	–17.668	0.012	–7.062	0.032
AK140218	6.516	0.002	14.123	0.037

a Values indicate the absolute fold-change between paired samples (adult to neonatal ratio, A/N) detected by microarray or qRT-PCR; negative value indicates down-regulation and positive value indicates up-regulation.

b p Value was calculated by the Student's T-test (paired).

### Genomic association of differentially expressed lncRNAs with protein-coding genes

**Figure 4:**
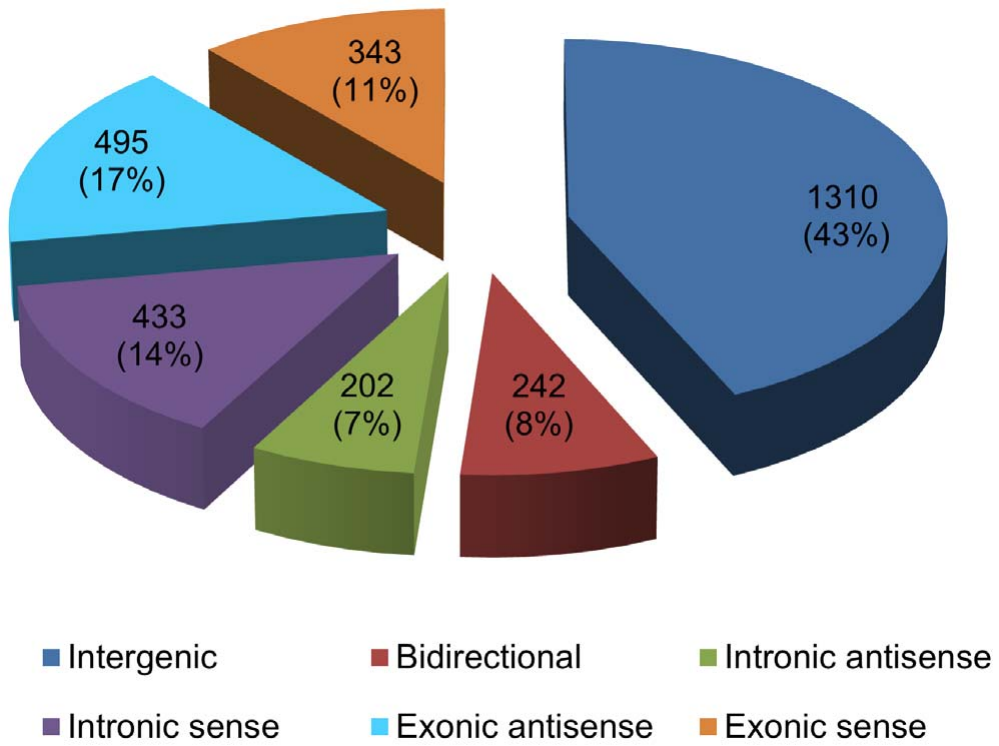
Annotation of genomic context of differentially expressed lncRNAs.

Previous studies show that lncRNAs often originate from complex transcriptional loci, in which the lncRNAs are coordinately transcribed with their associated protein-coding transcripts [33], [34]. A number of investigations have indicated that the exact nature of the genomic relationship between a lncRNA and its associated protein-coding gene usually has important functional consequences, often with the lncRNA regulating the expression of its protein-coding counterpart via epigenetic modification or transcriptional co-activation/repression [35]–[37]. To gain insight into the functional role of lncRNAs that are differentially expressed during testis development, we analyzed their genomic context based upon their orientation to local protein-coding genes. We categorized the relationship between lncRNAs and their associated protein-coding genes as exonic sense, intronic sense, exonic antisense, intronic antisense, bidirectional and intergenic according to our modified definition (see Materials and Methods). Of 3,025 differentially expressed lncRNAs, we identified 343 exonic sense, 495 exonic antisense, 433 intronic sense, 202 intronic antisense, 242 bidirectional and 1,310 intergenic lncRNAs (Figure 4, Table S6).

**Figure 5:**
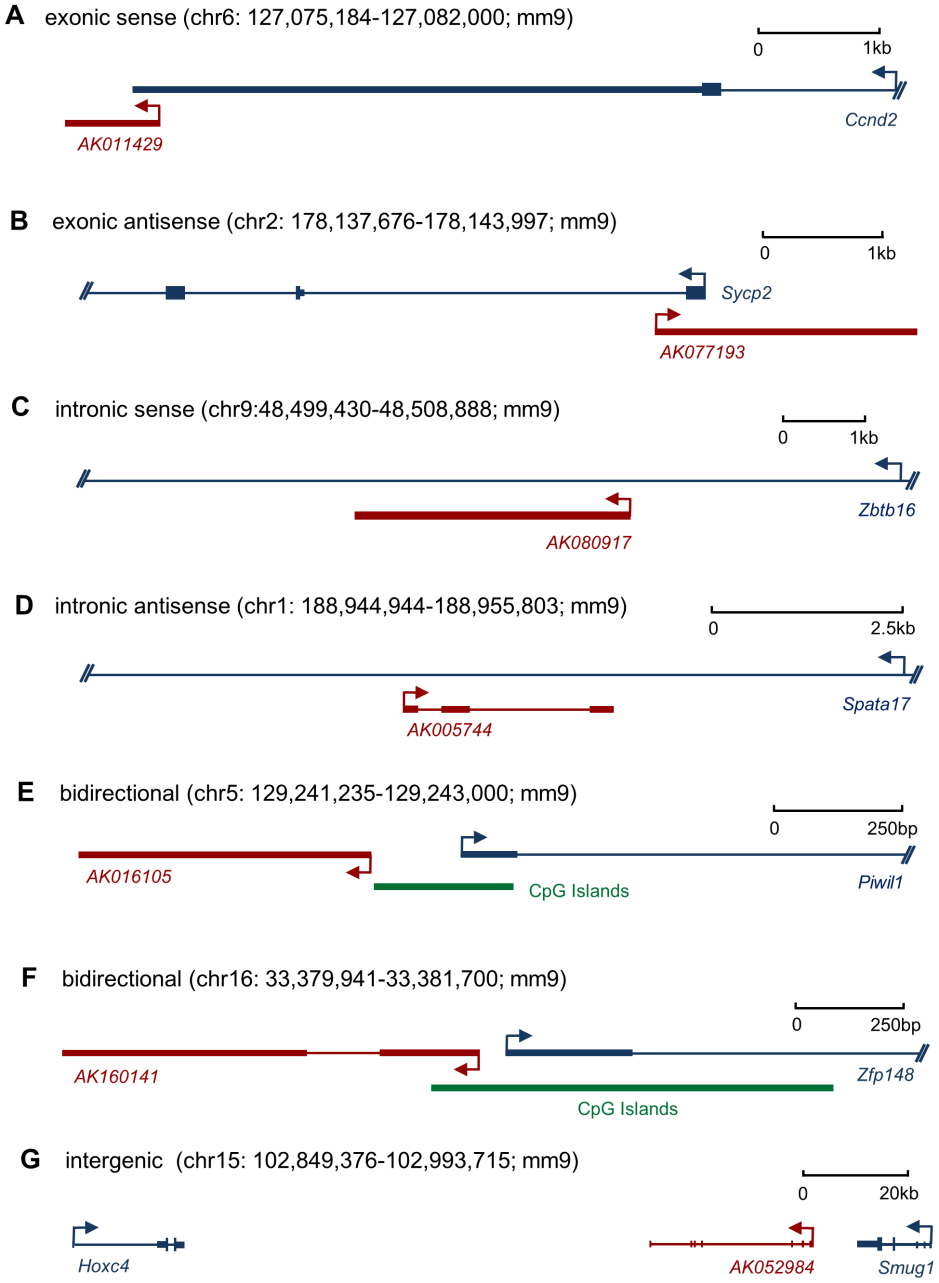
Differentially expressed lncRNAs share loci with spermatogenesis-related protein-coding genes. Examples of exonic sense (A), exonic antisense (B), intronic sense (C), intronic antisense (D), bidirectional (E and F), and intergenic (G) lncRNAs are shown. Each panel illustrates the organization of the lncRNA (red) to associated protein-coding genes (blue). CpG islands are indicated by green strips. Arrows indicate the direction of transcription.

**Exonic sense lncRNAs.** Transcriptional profiling has shown that transcription of sense lncRNAs that overlap with exons of protein-coding genes is pervasive throughout the mammalian genome, and many of these lncRNAs can be considered non-coding transcript variants of protein-coding genes [9]. Previous studies have demonstrated that exonic sense lncRNAs can regulate the expression of their associated protein-coding genes [38]. Here, we found 343 lncRNAs were exonic sense. For example, *AK011429*, a down-regulated lncRNA, was identified as an exonic sense transcript to cyclin D2 (*Ccnd2*) (Figure 5A). The expression profile of *AK011429* was positively correlated with that of *Ccnd2*. *Ccnd2* is highly expressed in spermatogonia and plays an important role in spermatogonial stem cell (SSC) self-renewal [39].

**Exonic antisense lncRNAs.** Exonic antisense transcripts are prevalent throughout the mammalian genome and are expressed at high levels in the testis [40], [41]. Numerous studies have demonstrated that they can regulate the expression of their protein-coding counterparts via a range of mechanisms, including chromatin remodeling, alternative splicing, translational promotion, translational interference and promoter targeting [42]. Within our data sets, we found that 495 of the differentially expressed lncRNAs were exonic antisense transcripts. For example, *AK077193* was an exonic antisense transcript to synaptonemal complex protein 2 (*Sycp2*) (Figure 5B), a spermatocyte-specific gene required for synaptonemal complex assembly and chromosomal synapsis during male meiosis [43]. Our data show that *AK077193* was up-regulated in adult testis and there was a positive correlation between expression of *AK077193* and *Sycp2.* Therefore, we speculated that *AK077193* was likely to regulate *Sycp2* expression.

**Intronic lncRNAs.** A class of long non-coding RNAs transcribed from intronic regions of protein-coding genes, including from sense and antisense strands, was recently identified by the Verjovski-Almeida group, and they have demonstrated that intronic lncRNAs reside in a large portion of mammalian transcriptional units [44], [45]. Previous studies indicated that intronic lncRNAs could regulate the expression of their host or neighboring genes via multiple mechanisms, including alternative splicing, microRNAs and RNA interference, transcriptional disruption and chromatin modification [46]. In the current study, we identified 433 intronic sense and 202 intronic antisense transcripts from differentially expressed lncRNAs. For instance, AK080917, a down-regulated lncRNA in adult testis, was identified as an intronic sense transcript from the region between introns 4 and 5 of Zinc finger and BTB domain containing 16 (Zbtb16) (Figure 5C). Zbtb16 has a high expression level in spermatogonial stem cells and is a key gene required for their maintenance [47]. A positive correlation between expression of AK080917 and Zbtb16 suggested that their function and/or regulation might be related. Another example is AK005744, which is specifically expressed in testis and up-regulated in adult testis. AK005744 is an intronic antisense transcript from the region between introns 6 and 7 of Spermatogenesis associated 17 (Spata17) (Figure 5D), a testis-specific gene involved in male germ cell apoptosis and strongly expressed in adult testis [48], [49]. The positive correlation between expression profiles of AK005744 and Spata17 indicated that there might be some regulatory relationships between them.

**Bidirectional lncRNAs.** A major organizational theme within mouse and human transcriptomes, which applies to approximately 10% of known protein coding genes, is the prevalence of bidirectional transcripts. These are pairs of transcription initiation sites from two different transcripts that are in close proximity (<1000 bp) but in the opposite orientation [50], [51]. Bidirectional genes usually share a common CpG island promoter [52], while bidirectional lncRNAs can promote or repress the expression of their neighboring protein-coding genes via epigenetic modification [53], [54]. Among the differentially expressed lncRNAs, we found that 242 possessed a bidirectional pair. As an example, we identified *AK016105* as a bidirectional pair to *Piwi-like homolog 1* (*Piwil1*), a testis-specific gene specifically expressed in spermatocytes and spermatids. *Piwil1* plays a central role during meiosis through piRNA-mediated repression of transposable elements and translation regulation [55]. In addition, their common promoter region has a CpG island (Figure 5E). High expression levels of *AK016105* in adult testis were concordant with the expression profile of *Piwil1*. Another example, *AK160141*, was identified as a bidirectional pair to *Zinc finger protein 148* (*Zfp148*), which is strongly expressed in neonatal testis and is required for normal development of male germ cells [56]. This bidirectional pair also shares a common CpG island promoter (Figure 5F). Our data showed that *AK160141* was weakly expressed in neonatal testis. Therefore, it exhibited a discordant expression profile with *Zfp148*.

**Intergenic lncRNAs.** Genome-wide analysis in eukaryotes other than human and mouse has shown that long intergenic non-coding RNAs (lincRNAs) represent a large portion of the non-coding genes, for example in zebrafish, worm, and *Arabidopsis* [10], [26], [57], [58]. Emerging evidence supports the view that lincRNAs play important roles in many fundamental biological processes, such as pluripotency and differentiation of embryonic stem cells, brain development and limb development, and are also involved in certain diseases, especially cancer [59], [60]. Although, lincRNAs are poorly conserved across species [59], an increasing number of studies have demonstrated that they can modulate the expression of their neighboring protein-coding genes or other target genes scattered across the genome via directly recruiting histone-modifying enzymes to chromatin [35], [61]. We identified 1,310 lincRNAs among the differentially expressed lncRNAs. In particular, a large number of lincRNAs were transcribed from *HOX* loci and were spatially expressed along developmental axes. These lincRNAs possess unique sequence motifs, and their expression can control the expression of neighboring *HOX* genes by affecting chromatin signature [35]. For example, *AK052984*, a lincRNA highly expressed in adult testis, was transcribed from the intergenic region between *Hoxc4* and *Smug1*. *Hoxc4* is required for spermatogonial stem cell self-renewal and is highly expressed in neonatal testis [62]. The expression of *AK052984* was clearly negatively correlated with the expression of *Hoxc4*. Therefore, we speculated that *AK052984* was likely to affect *Hoxc4* expression.

### Some differentially expressed lncRNAs overlap microRNAs

**Figure 6:**
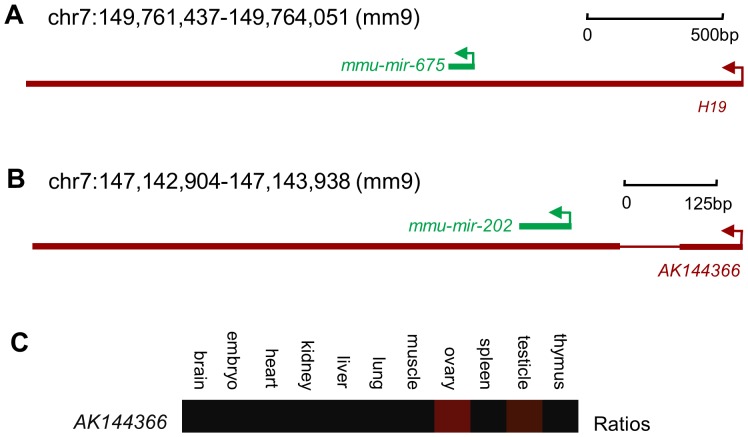
Examples of LncRNAs that overlap with microRNAs. Each panel illustrates the lncRNA (red) transcription initiation direction (indicated by an arrow) relative to an annotated microRNA (green). (A) *H19* overlaps with *mmu-mir-675.* (B) *AK144366* overlaps with *mmu-mir-202.* (C) Tissue expression pattern of *AK144366*, according to the Affymetrix Exon Tissues Track in the UCSC Genome Browser. Red indicates high level of expression.

Small noncoding RNAs, such as microRNAs, can be processed from long primary ncRNAs [63]. It is well-known that mature microRNAs can regulate the stability and fate of their target protein-coding RNAs via binding to the 3’-UTR, 5’-UTR or coding region [64]. Recent studies show that lncRNAs can also be targeted by microRNAs [65]. To explore the possibility that some lncRNAs that are differentially expressed during testis development might also act as primary transcripts for small RNAs, we systematically searched for genomic overlap between differentially expressed lncRNAs and known microRNAs. We found 20 lncRNAs overlapped annotated microRNAs (Table S7). For example, we detected that *H19*, a well-known paternally imprinted gene, was the most significantly down-regulated lncRNA in the adult testis and contained *mmu-mir-675* (Figure 6A). A recent study has demonstrated that *H19* can indeed be processed *in vivo* to give rise to the 23 nt-long miR-675 miRNA and that this ability is conserved in humans and mice [66]. *AK144366*, a lncRNA down-regulated in neonatal testis, contains *mmu-mir-202* within its second exon (Figure 6B). This direct overlap indicates that *AK144366* is likely to be processed into and function via *mmu-mir-202*, and indeed *mmu-mir-202* expression is up-regulated in neonatal testis and down-regulated in adult testis [67]. Furthermore, the expression pattern of *AK144366* was concordant with that of *mir-202*, according to Affymetrix Exon Tissues Track (hosted in the UCSC Genome Browser, http://genome.ucsc.edu/) (Figure 6C). This further indicated that *AK144366* might be the primary precursor to *mmu-mir-202*.

### LncRNA-associated protein-coding genes are more likely to function in transcription-related processes

As previously mentioned, lncRNAs are often coordinately transcribed with their associated protein-coding transcripts and can regulate the expression of their adjacent or overlapping protein-coding genes in multiple ways. To some extent, the function of lncRNAs may be reflected through their associated protein-coding genes. Therefore, gene ontology (GO) term enrichment analysis of associated protein-coding genes may provide insight into the function of lncRNAs. We submitted a list of 3,275 protein-coding genes associated with differentially expressed lncRNAs to the Database for Annotation, Visualization and Integrated Discovery (DAVID) for GO term enrichment analysis and 2,940 genes had functional annotation in DAVID. The most relevant GO terms were enriched in transcription-related processes and some key transcriptional regulator genes involved in spermatogenesis occurred in the “regulation of transcription” gene list, and including *Ovol1*, *Ovol2, Lhx1*, *Sox3*, *Sox9*, *Plzf* (*Zbtb16*), *Tfam*, *Notch1* and *Tnp1* (Table 5, Table S8). It is indicated that lncRNAs were likely to perform their function at transcriptional levels by regulating transcription-related genes. In contrast, GO terms related to testis development or spermatogenesis were poorly represented and enriched (Table 5). It is noteworthy that our results showed extremely similar trends to those observed in a previous investigation [26]. Although there was little GO term enrichment for testis development and spermatogenesis-related genes, we still found that approximately 2.5% of genes (69 of 2,940) had associated GO terms relating to testis development or spermatogenesis, included some well-known spermatogenesis genes, such as *Plzf* (*Zbtb16*), *Kit*, *Wt1*, *Notch1*, *Piwil1*, *Sycp2*, *Prm1* and *Prm2* (Table S9).

**Table 5:** Representative results of GO term encrichment for lncRNAs associated protein-coding genes.

GO term	Count^a^	Fisher Exact p-value^b^
Transcription factor activity (GO:0003700)	187	1.5E-12
Regulation of transcription (GO:0006355)	441	4.8E-12
Positive regulation of transcription (GO:0045893)	126	3.4E-11
Transcription (GO:0006351)	358	7.8E-11
Negative regulation of transcription (GO:0045892)	84	7.3E-5
Germ cell development (GO:0007281)	24	0.023
Male gonad development (GO:0008584)	12	0.024
Gamete generation (GO:0007276)	60	0.078
Spermatogenesis (GO:0007283)	43	0.26

a The gene members, which belong to an annotation term.

b Fisher Exact p-value representing the degree of enrichment of the GO term.

### Most differentially expressed lncRNAs exhibit epigenetic modification marks and are preferentially expressed in a tissue-specific manner

Epigenetic modifications, such as DNA methylation and histone modification, are important regulators in various biological processes, including testis development and spermatogenesis. A large number of protein-coding genes are regulated during testis development and spermatogenesis via epigenetic mechanisms [68]. Similar to protein-coding genes, most lncRNAs are transcribed by RNA polymerase II and transcribed products have typical hallmarks of Pol II, such as a 5’ cap and poly(A) tail [10]. Furthermore, many lncRNAs are expressed in specific cell and/or tissue-types and during specific developmental stages. These suggest that lncRNAs may be regulated epigenetically in a similar way to protein-coding genes. This is also supported by previous studies showing that lncRNAs promoters are subject to purifying selection, are on average more conserved than promoters of protein-coding genes, and are associated with chromatin marks [9], [69]–[71]. Therefore, to investigate whether differentially expressed lncRNAs are subject to epigenetic regulation in the adult testis, we carried out an analysis to identify promoters with high CpG content (HCG), histone H3 lysine 4 trimethylation (H3K4me3) and histone H3 lysine 27 trimethylation (H3K27me3) level.

In mammals, DNA methylation occurs at cytosines within the context of the CpG dinucleotide, which are frequently found in short genomic regions including gene promoters [72]. Mammalian gene promoters can be classified into distinct categories based on their CpG dinucleotide content. HCG promoters are typically non-tissue-specific and frequently regulate housekeeping genes or genes with complex expression patterns and approximately 64% of protein-coding genes have an HCG promoter (HCP) [73]. Therefore, we first identified the promoters of lncRNAs based on CpG content and found that only 23% of differentially expressed lncRNAs (689 out of 3,025) were associated with HCPs and that the CpG observed-to-expected ratio (CpG O/E) ranged from 0.61 to 1.43 (Table S10). This result indicated that a large proportion of differentially expressed lncRNAs were likely to function in a tissue-specific manner, as suggested by previous studies [13].

H3K4me3 and H3K27me3 regulate gene expression and therefore play key roles in multiple aspects of development [74]. Previous studies indicate that H3K4me3 is generally associated with activation of transcription, while H3K27me3 is closely associated with transcriptional repression [75]. To investigate whether H3K4me3 and H3K27me3 can affect the expression of differentially expressed lncRNAs, we assessed the levels of H3K4me3 and H3K27me3 on lncRNA promoters in adult mouse testis. We found that 73% of up-regulated lncRNAs (1,442 out of 1,963) possessed H3K4me3 marks on their promoters; in contrast, only 32% of down-regulated lncRNAs (338 out of 1062) have H3K4me3 marks (Table S10). The correlation between promoter H3K4me3 marks and lncRNA expression was consistent with that H3K4me3 involved in transcriptional activation. For H3K27me3, only 8% of down-regulated lncRNAs promoters (86 out of 1,062) were marked by H3K27me3, while 4% of up-regulated lncRNAs (71 out of 1,963) had H3k27me3-positive promoters (Table S10). Although a minority of differentially expressed lncRNAs had H3k27me3 marks on their promoters, these results were still consistent with the negative regulatory effect of H3k27me3. In addition, many promoters with H3K4me3 modification are simultaneously marked with H3K27me3 and these so-called “bivalent” domains mark developmentally-associated genes whose expression is thought to be “poised” for activation or repression during development [76]. We found that only approximately 5% of differentially expressed lncRNAs (138 out of 3,025) had bivalent promoters. However, nearly 88% of H3K27me3-positive lncRNAs promoters (138 out of 157) were also marked by H3K4me3, which is similar to previous reports for protein-coding genes [77].

In addition, previous studies revealed that HCPs are almost always decorated with H3K4me3 and prefer to regulate ubiquitous housekeeping genes [76]. Here, we found 94% of differentially expressed lncRNAs with a HCP were associated with H3K4me3 (649 out of 689), which further indicated that these lncRNAs were extremely likely to be ubiquitously expressed as housekeeping genes and that the majority of differentially expressed lncRNAs perform tissue-specific functions.

We concluded that the majority of lncRNAs differentially expressed during testis development were likely to be expressed in a tissue-specific pattern, and that they could be regulated by epigenetic modification, similar to protein-coding genes.

### Most differentially expressed lncRNAs contain evolutionarily conserved elements

Over evolutionary time, the purifying selection of functional genomic elements results in the presence of sequences that exhibit high levels of conservation across multiple species, providing a useful indicator of function, for example, functional protein-coding sequence is often highly conserved. LncRNA sequences generally exhibit low conservation [14]; however, recent studies have revealed that a large number of lncRNAs contain a short highly conserved sequence in exons, especially in the exon-intron boundaries [78], [79]. Therefore, we identified this conserved region (termed PhastCons elements) within differentially expressed lncRNAs that are strongly conserved across species (see Materials and Methods). We found that 70% of differentially expressed lncRNAs overlapped PhastCons elements (2,129 out of 3,025), 93% of which (1,986 out of 2,129) possessed ≥20 PhastCons bases (Table S11). Hence, the majority of lncRNAs differentially expressed during testis development exhibited evolutionary conservation of primary sequences. This result was consistent with previous reports [12].

## Discussion

In recent years, increasing evidence have demonstrated that lncRNAs play key roles in the development of various tissues, such as brain [25], retina [80], mammary gland [81], heart [82], [83], and inner ear [84]. However, very few studies have been conducted on the potential roles of lncRNAs in mammalian testis development. Investigations into the molecular mechanisms of testis development and spermatogenesis, have mostly focused on protein-coding genes and small non-coding RNAs (miRNAs and piRNAs). Therefore, our understanding of lncRNA function in mammalian testis development is still extremely poor. For this reason, in the current study, we took advantage of the high throughput feature of microarrays to investigate lncRNAs expression profiles of neonatal and adult mouse testes. This is the first systematic expression profiling study of lncRNAs during post-natal development of the mammalian testis employing genome-wide techniques.

lncRNAs are generally expressed at lower levels than protein-coding genes and are more likely to display a tissue-specific pattern of expression [13], [85], [86]. To compare the overall expression status of lncRNAs and mRNAs in mouse testis, we employed microarrays containing probes for both lncRNAs and mRNAs to simultaneously detect the expression of lncRNAs and mRNAs. Consistent with previous reports, we found that only 56% of lncRNAs were expressed above background in mouse testis, while this number reached 82% for protein-coding genes. This finding suggested that lncRNAs tended to be expressed at lower levels and in a testis-specific manner and that they might represent cryptic signals that mainly fulfill regulatory functions in the control of complex testis developmental processes and spermatogenesis. In addition, we found that nearly all mitochondrial genome-derived lncRNAs on the microarray could be detected above background. This finding indicated that mitochondrial genome-derived lncRNAs were relatively abundant in testis, and that lncRNAs might be key contributors to mitochondria-mediated regulation of spermatogenesis; mitochondria are known to play an important role in spermatogenesis, such as meiosis, quality control through apoptosis and sperm motility. We also found that the number of lncRNAs transcribed from the X chromosome is much larger in the down-regulated group than the up-regulated group. It is indiatded that X chromosome inactivation during spermatogenesis could affect the expression of lncRNA similar to protein-coding genes.

In this study, we identified 3,025 lncRNAs that were significantly differentially expressed between neonatal and adult mouse testis. The dynamic change of lncRNA expression during testis post-natal development further indicated that lncRNAs might play significant biological roles in testis development and spermatogenesis. Unlike protein-coding gene or microRNAs, the function of lncRNAs cannot currently be inferred from sequence or structure. Therefore, to date, most studies have predicted function via genomic association of lncRNAs with protein-coding genes because lncRNAs often regulate the expression of their overlapping or neighboring protein-coding genes [87]. Therefore, we analyzed the genomic context of differentially expressed lncRNAs and classified them based on their genomic relationship with protein coding genes as exonic sense, intronic sense, exonic antisense, intronic antisense, bidirectional and intergenic. We found that all six categories of differentially expressed lncRNA could be examined. Strikingly, we found that nearly 43% of differentially expressed lncRNAs (1,310 out of 3,025) were long intergenic non-coding RNAs (lincRNAs). This result indicated that lincRNAs were more abundant in testis relative to other classes of lncRNAs that overlap with protein coding genes, and that they might be the major contributor to the regulatory roles mediated by lncRNAs. Interestingly, a recent study reported that 78% of all defined lincRNAs (4662 in total) exhibited tissue-specific expression patterns relative to protein-coding genes and almost a third of lincRNAs were specific to testis [59]. Combined with our findings, this suggested that lincRNAs are abundant in testis and can be considered as a new class of RNA in the testis, like piRNA [88]. To further define which biological processes lncRNAs may be involved in, we tested for GO enrichments in the set of protein-coding genes associated with lncRNAs in a genomic context. We found that protein-coding genes that overlap or are adjacent to lncRNAs were inclined to be enriched in transcription related processes. Notably, our result was consistent with previous reports on brain development and embryonic development [26], [89]. Some lncRNAs may, therefore, function as “indirect regulators” of transcription by regulating protein-coding genes responsible for transcription.

Recent studies have demonstrated that lncRNAs can be regulated by epigenetic modification of their promoter regions, such as DNA methylation and histone modification, in a manner similar to protein-coding genes. The dynamic lncRNA expression profiles during testis development prompted us to investigate promoter characteristics related to epigenetic modification, including whether promoters are high CpG content promoters (HCP), and determining levels of H3K4me3 and H3K27me3. We found that only 23% of differentially expressed lncRNAs were defined as HCPs. HCPs are generally associated with ubiquitously expressed “housekeeping” genes; therefore, we inferred that the majority of differentially expressed lncRNAs were likely to be expressed in a testis-specific manner. Our finding was consistent with previous observations showing that most lncRNAs usually exhibit tissue-specific expression patterns [59], [86]. In addition, we found that there was also a strong correlation between histone modification, including H3K4me3 and H3K27me3, and the expression level of the associated lncRNAs, similar to that observed for protein-coding genes. For instance, in adult mouse testis, we found that almost 73% of up-regulated lncRNAs and only 32% of down-regulated lncRNAs were marked at their promoters by H3K4me3. This result was consistent with previous observations showing that the H3K4me3 mark is strongly correlated with gene activation. We also found that almost all lncRNAs with HCPs often exhibited the H3K4me3 mark simultaneously, very similar to previous reports for protein-coding genes. Clearly, this sub-class of promoters preferentially regulates ubiquitous housekeeping genes further indicated that the majority of differentially expressed lncRNAs might be specifically expressed in testis. It is noteworthy that we found a small percentage of differentially expressed lncRNAs marked by both H3K4me3 and H3K27me3 in adult testis. We speculate that these lncRNAs may be important developmental regulators because developmental regulator genes often carry bivalent histone modifications of both H3K4me3 and H3K27me3 [77].

In addition, we found that approximately half the differentially expressed lncRNAs were spliced according to annotation from the UCSC Mouse Genome Browser database (mm9), similar to most protein-coding genes (Table S12). This indicated that the majority of lncRNAs differentially expressed during testis development were transcribed by RNA polymerase II (Pol II) because pre-mRNA splicing is frequently coupled to transcription mediated by Pol II [90]. Therefore, it was not surprising that lncRNAs could be regulated via similar epigenetic modifications to those that regulate protein-coding genes.

Most lncRNAs generally exhibit poor primary sequence conservation; however, recent investigations have found short, highly conserved regions in lncRNA primary sequences. Indeed, we found that nearly 70% of differentially expressed lncRNAs contain PhastCons conserved elements. An explanation for the low sequence conservation of lncRNA sequences may be that they do not require very much nucleotide sequence conservation to maintain their functionality. Previous studies have demonstrated that a large number of lncRNAs can bind histone modification complexes, such as polycomb repressive complex 2 (PRC2), then guide these complexes to specific sites and cause the silencing of target genes via histone modification [91]. It is possible that similar secondary structures formed by lncRNAs with distinct sequence contribute to the specificity of interactions with the same protein partners. For example, *Xist* and *HOTAIR*, two well-known functionally annotated lncRNAs, bind to PRC2. Both have similar, short GC-rich stem-loop RNA motifs that are required for recruitment of PRC2 [92], [93]. Therefore, to maintain normal function, these lncRNAs may only need to conserve short stretches of sequence that form similar secondary structures. In contrast, protein coding genes are under intense selection restraints due to the need to maintain correct amino acid coding and an open reading frame.

In summary, this is the first systematic study to examine the expression profiles of lncRNAs in mammalian testis on a genome-wide scale. The dynamic expression profiles and feature analyses, including genomic context, epigenetic modification of promoters and evolutionary conservation, indicate that lncRNAs may play important roles in post-natal development of the mammalian testis. Although due to technical restriction of microarray, it is not possible to profile entire transcriptome of lncRNAs in this study; our results still provide a solid foundation for the identification and characterization of key lncRNAs involved in testis development or spermatogenesis.

## Materials and Methods

### Animals

Twenty one neonatal (6-day-old) and six adult (8-week-old) male C57BL/6 mice were purchased from SLAC Laboratory Animal Co., Shanghai, China. Mice in each age group were divided into three groups to provide three biological replicates for microarray analysis. All procedures involving animals were approved by Institutional Animal Care and Use Committee (IACUC) at Shanghai Jiao Tong University, Shanghai, China [SYXK (Shanghai 2007-0025)], and were conducted in accordance with the National Research Council Guide for Care and Use of Laboratory Animals.

### RNA isolation and labeling

RNA derived from mouse testes was purified using Trizol Reagent (Invitrogen) and treated with DNase I (Fermentas). The quantification and quality of RNA samples were assessed using a NanoDrop ND-1000 Spectrophotometer (Thermo Scientific). RNA integrity and genomic DNA contamination were examined by denaturing agarose gel electrophoresis. Double-stranded cDNA was synthetized from RNA using an oligo dT primer and a Superscript Double-Strand Synthesis Kit (Invitrogen, 11917-020). cDNA was labeled with Cy3 using the Quick Amp labeling kit (Agilent). Labeled cDNA quality assessment and quantification were performed using a NanoDrop-1000 Spectrophotometer (Thermo Scientific).

### Microarray expression analysis

Labeled cDNA was hybridized to the Mouse Stringent LncRNA Microarray (Arraystar) using Agilent’s SureHyb Hybridization Chambers according to the One-Color Microarray-Based Gene Expression Analysis protocol (Agilent Technology). After hybridization and washing, slides were scanned with the Agilent DNA microarray scanner (G2505B) using the recommended settings. The resulting text files, extracted from Agilent Feature Extraction Software (version 10.5.1.1), were imported into the Agilent GeneSpring GX software (version 11.0) for quantile normalization and background correction. Probe level files and gene summary files were produced. Differentially expressed lncRNAs and mRNAs were identified through Absolute fold change and P values were calculated using Student's T-test (paired). An Absolute fold change of ≥5.0 and a P value of ≤0.05 were selected as thresholds for significant differential expression. Raw and processed microarray data have been deposited in the National Center for Biotechnology Information (NCBI) Gene Expression Omnibus (GEO) and are accessible through (GEO) series accession number, GSE43442.

### Quantitative real-time PCR

Quantitative real-time PCR (qRT-PCR) was performed according to the manufacturer’s protocols (Roche). The qRT-PCR reactions were performed on a 96-well plate in triplicate. Each reaction consisted of a 25 µL mixture containing 12.5 µL 2×FastStart Universal SYBR Green Master (Roche), 0.3 µM forward and reverse primer and 40 ng cDNA. qRT-PCR amplifications were performed using the ABI PRISM 7500 Sequence Detection System (Applied Biosystems). Amplification conditions were as follows: 10 min at 95°C to activate the FastStart Taq DNA polymerase followed by 40 cycles of 15 s at 95°C and 30 s 60°C. A dissociation curve was drawn to ensure the validity of each specific PCR product. The qRT-PCR was repeated three times. The relative expression of genes was calculated based on the 2^−ΔΔCt^ method using the mouse housekeeping GAPDH gene as an endogenous control [94]. Differences in expression levels between two groups were evaluated using Student's t-test (paired). Primers used are shown in Table S12. The primers were designed using Primer Premier 5.0 and checked using Primer-BLAST searches to avoid cross-amplification. Amplification efficiency was evaluated via standard curve analysis.

### LncRNA genomic context analysis

We determined the genomic context of lncRNAs in relation to protein-coding genes according to a protocol that was updated for this study (Figure S3) [12]. In summary, exonic sense and antisense lncRNAs were defined where the corresponding transcript was mapped to the positive and opposite strand, respectively, of a RefSeq-annotated exon [includes 5′-untranslated region (UTR), coding exon, and 3′-UTR]. Intronic sense and antisense lncRNAs were defined where the corresponding transcript was mapped to the positive and opposite strand, respectively, of an intron of a protein-coding gene. Bidirectional lncRNAs were defined where the corresponding transcript was oriented head-head to a protein-coding gene at a distance of <1000 bp. Intergenic lncRNAs were defined where the corresponding transcript was located within an intergenic region and no overlapping or bidirectional coding transcripts were nearby.

### Gene Ontology (GO) term analysis of lncRNAs associated with protein-coding genes

Information on Gene Ontology functions of differentially expressed lncRNAs associated with protein-coding genes was obtained from the Database for Annotation, Visualization and Integrated Discovery v6.7 (DAVID) (http://david.abcc.ncifcrf.gov/) [95]. Statistically over-represented GO terms in the biological process and molecular function categories were obtained by applying a Fisher's exact p-value cutoff <0.05, and correcting for multiple testing with the Benjamini false discovery rate. The mouse genome was used as the reference set.

### LncRNA promoter analysis

High CpG content promoters (HCPs) were identified as described previously [76]. Briefly, transcripts in a 500 bp interval, within −0.5 to +2 kb of the transcription start site (TSS), with a GC fraction≥0.55 and a CpG observed-to-expected ratio (CpG O/E) ≥0.6 were classified as HCPs. The CpG O/E was calculated as described previously [96]. H3K4me3 and H3K27me3 status of promoter regions (−0.5 kb to +2 kb of the TSS) was obtained from publicly available H3K4me3 (UCSC accession: wgEncodeEM002489) and H3K27me3 (UCSC accession: wgEncodeEM002723) ChIP-Seq data previously generated from testes of 8-week-old mice, respectively. The peaks score corresponding to a promoter region was used to evaluate the level of H3K4me3 and H3K27me3 within the promoter.

### Evolutionary conservation analysis of lncRNAs

Conservation of each LncRNA was determined by intersecting its sequence with those of genome-wide PhastCons elements. The number of total bases annotated as PhastCons elements was used to evaluate the evolutionary conservation. The PhastCons program uses genome alignments across 30 vertebrate species (30-way) to identify conserved genomic regions based upon a phylogenetic hidden Markov model [97].

## Supporting Information

Figure S1 **Hierarchical clustering of mRNAs differentially expressed in neonatal and adult mouse testis.** A hierarchical clustered heat map showing the log2 transformed expression values for differentially expressed lncRNAs (absolute fold-change ≥5; P≤0.05) between neonatal (N) and adult (A) mouse testes. The intensity of the color scheme is calibrated to the log2 expression values such that red refers to higher transcript abundance and blue refers to lower transcript abundance. The bar code on the right represents the color scale of the log 2 values. Each column represents the data from one of three biological replicates of each sample.
(PPT)

Figure S2 **Relative chromosomal distribution of up and down-regulated lncRNAs.** Chromosomes are on the X axis, and the distribution ratio is on the Y axis. Vertical bands show the ratio (up or down-regulated probe number/total probes number) of up and down-regulated lncRNAs derived from each chromosome.
(PPT)

Figure S3 **Genomic organization of lncRNAs and their associated protein-coding genes.** Schematic diagram illustrating the six categories of genomic association of lncRNAs (orange) with protein-coding genes (blue). Transcription initiation direction is indicated by an arrow (black).
(PPT)

Table S1 **LncRNAs expressed in mouse testis above background.**
(XLS)

Table S2 **LncRNAs differentially expressed in neonatal and adult mouse testis.**
(XLS)

Table S3 **mRNAs expressed in mouse testis above background.**
(XLS)

Table S4 **mRNAs differentially expressed in neonatal and adult mouse testis.**
(XLS)

Table S5 **Chromosome distribution of expressed lncRNAs.**
(XLS)

Table S6 **Annotation for genomic context of differentially expressed lncRNAs.**
(XLS)

Table S7 **Differentially expressed lncRNAs overlapping with annotated microRNAs.**
(XLS)

Table S8 **List of lncRNAs associated protein-coding genes that enriched in transcription-related GO terms.**
(XLS)

Table S9 **List of lncRNAs associated protein-coding genes that enriched in testis development or spermatogenesis-related GO terms.**
(XLS)

Table S10 **Epigentic status in promoter of differentially expressed lncRNAs in adult testis.**
(XLS)

Table S11 **Evolutionarily conserved elements in differentially expressed lncRNAs.**
(XLS)

Table S12 **List of primers used in the validation of microarray results by Q-PCR.**
(XLS)
