# Supplementary figures and images for: Long Non-Coding RNA Expression Profiling of Mouse Testis during Postnatal Development
Source: PLoS One. 2013 Oct 10;8(10):e75750. doi: 10.1371/journal.pone.0075750 (PMC3794988; doi:10.1371/journal.pone.0075750)

## Slide 1
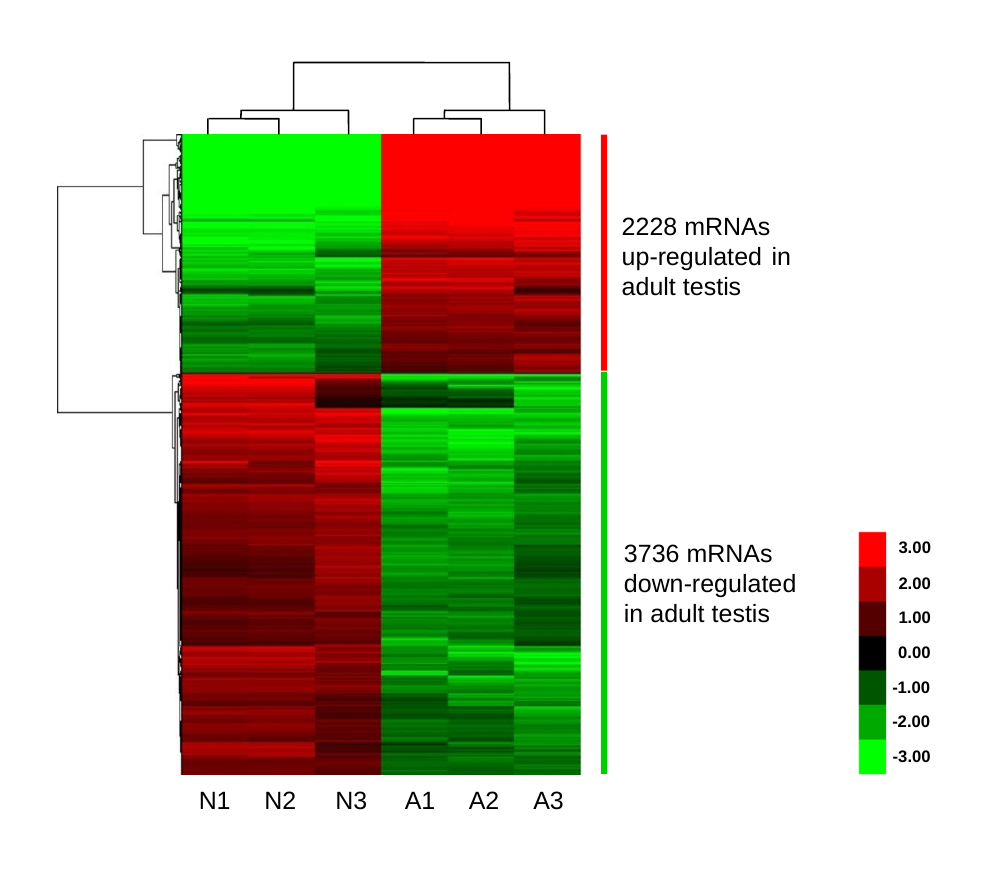

N1
N2
N3
A1
A2
A3
2228 mRNAs
up-regulated in adult testis
3736 mRNAs
down-regulated in adult testis
3.00
2.00
1.00
0.00
-1.00
-2.00
-3.00

Supplement: Figure S1 — Hierarchical clustering of mRNAs differentially expressed in neonatal and adult mouse testis. A hierarchical clustered heat map showing the log2 transformed expression values for differentially expressed lncRNAs (absolute fold-change ≥5; P≤0.05) between neonatal (N) and adult (A) mouse testes. The intensity of the color scheme is calibrated to the log2 expression values such that red refers to higher transcript abundance and blue refers to lower transcript abundance. The bar code on the right represents the color scale of the log 2 values. Each column represents the data from one of three biological replicates of each sample. (PPT) [file pone.0075750.s001.ppt]

## Slide 1
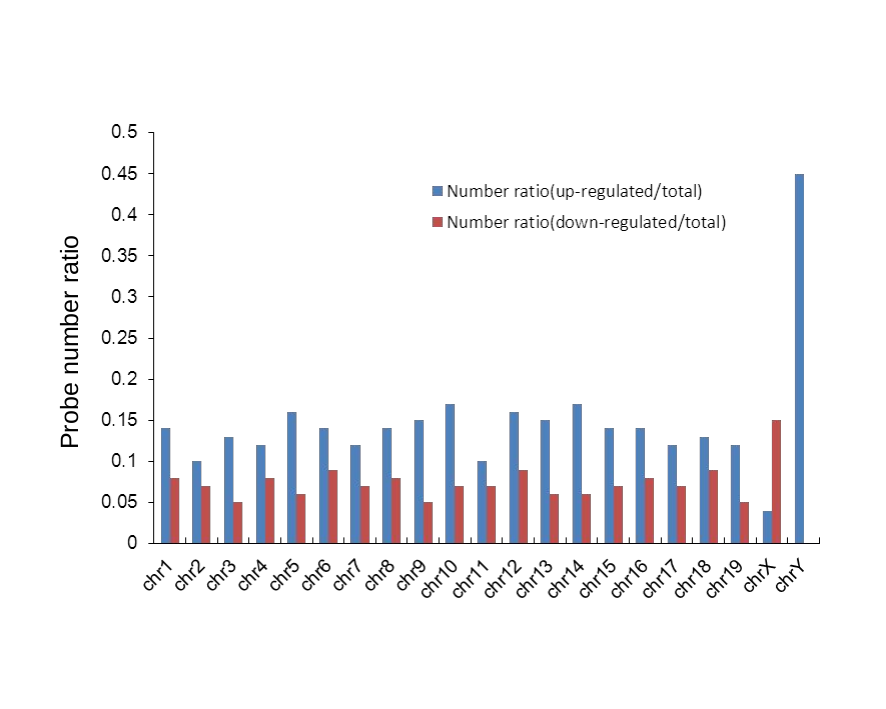

Probe number ratio

Supplement: Figure S2 — Relative chromosomal distribution of up and down-regulated lncRNAs. Chromosomes are on the X axis, and the distribution ratio is on the Y axis. Vertical bands show the ratio (up or down-regulated probe number/total probes number) of up and down-regulated lncRNAs derived from each chromosome. (PPT) [file pone.0075750.s002.ppt]
