# Supplementary material for: Long Non-Coding RNA Expression Profiling of Mouse Testis during Postnatal Development
Source: PLoS One. 2013 Oct 10;8(10):e75750. doi: 10.1371/journal.pone.0075750 (PMC3794988; doi:10.1371/journal.pone.0075750)
Supplement: Figure S3 — Genomic organization of lncRNAs and their associated protein-coding genes. Schematic diagram illustrating the six categories of genomic association of lncRNAs (orange) with protein-coding genes (blue). Transcription initiation direction is indicated by an arrow (black). (PPT) [file pone.0075750.s003.ppt]

## Slide 1
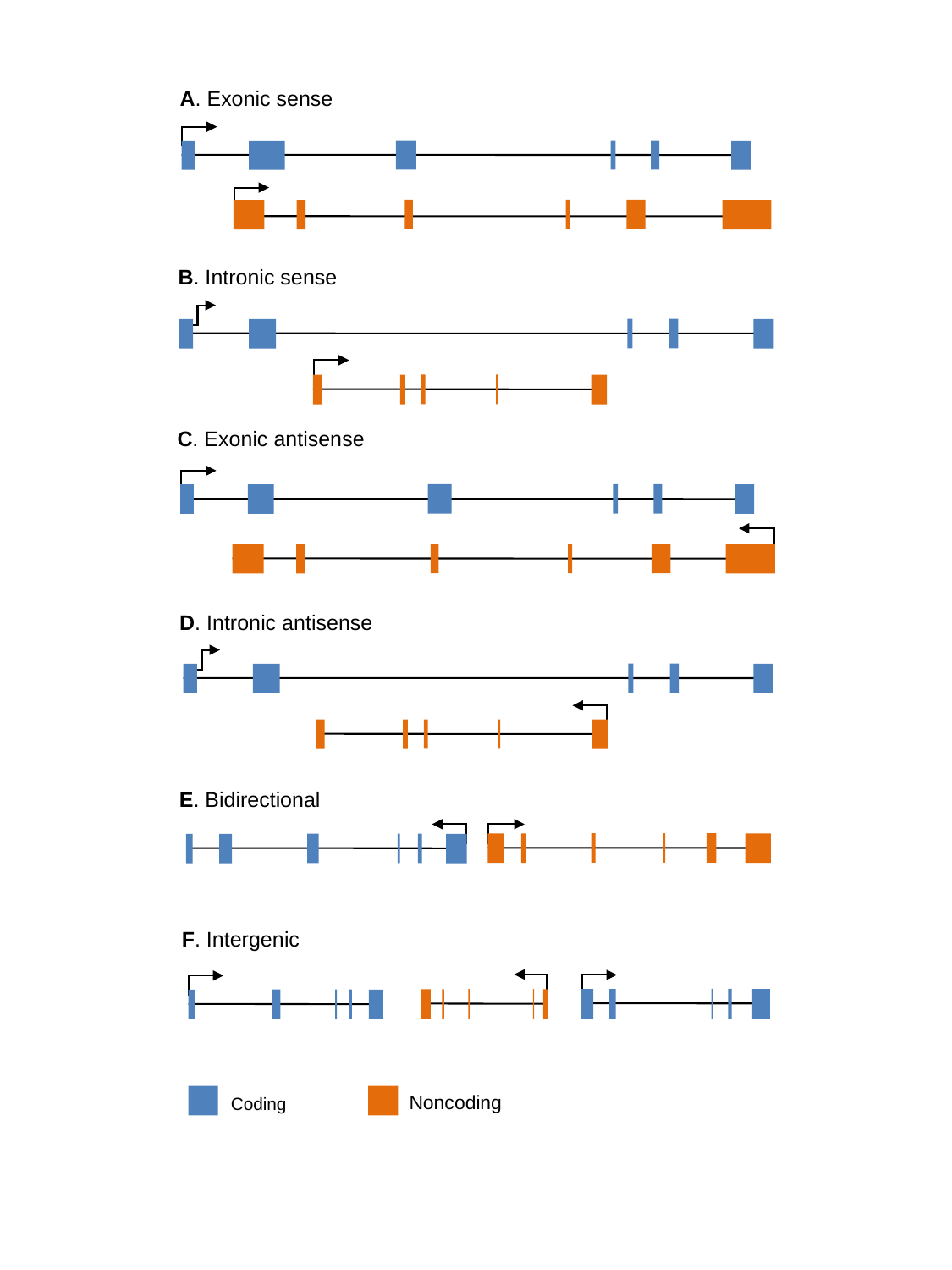

A. Exonic sense
B. Intronic sense
C. Exonic antisense
D. Intronic antisense
E. Bidirectional
F. Intergenic
Noncoding
Coding
